# Th17 Cells in Type 1 Diabetes: Role in the Pathogenesis and Regulation by Gut Microbiome

**DOI:** 10.1155/2015/638470

**Published:** 2015-12-30

**Authors:** Yangyang Li, Yu Liu, Cong-Qiu Chu

**Affiliations:** ^1^Department of Endocrinology, The Second Hospital of Jilin University, Changchun, Jilin 130041, China; ^2^Division of Arthritis and Rheumatic Diseases, Oregon Health & Science University and VA Portland Health Care System, Portland, OR 97239, USA

## Abstract

Type 1 diabetes (T1D) is an autoimmune disease which is characterized by progressive destruction of insulin producing pancreatic islet *β* cells. The risk of developing T1D is determined by both genetic and environmental factors. A growing body of evidence supports an important role of T helper type 17 (Th17) cells along with impaired T regulatory (Treg) cells in the development of T1D in animal models and humans. Alteration of gut microbiota has been implicated to be responsible for the imbalance between Th17 and Treg cells. However, there is controversy concerning a pathogenic versus protective role of Th17 cells in murine models of diabetes in the context of influence of gut microbiota. In this review we will summarize current knowledge about Th17 cells and gut microbiota involved in T1D and propose Th17 targeted therapy in children with islet autoimmunity to prevent progression to overt diabetes.

## 1. Introduction

In 2005, T helper type 17 (Th17) cells were first identified as a distinct T helper cell lineage [1, 2]. The discovery of Th17 subset not only changes the classical Th1/Th2 paradigm in T cell immune responses, but also provides us with new insights into the pathophysiological process in several autoimmune diseases [3]. Type 1 diabetes (T1D), one of the most prevalent autoimmune diseases which were previously thought to be mediated by Th1 cells, is now establishing a connection with Th17 cells [4]. Exploration of Th17 cells in T1D pathogenesis has attracted more and more attention nowadays. Here, we briefly review the findings that led to the identification of Th17 cells, their differentiation and functions, and interaction between Th17 and T regulatory (Treg) cells and integrate current knowledge about the influence of microbiota on Th17 cells and Treg cells in T1D. Finally, several approaches are being explored for intervention to block interleukin- (IL-) 17 activity or suppress Th17 differentiation or restore balance of Treg and Th17 cells. Manipulation of gut microbiota is an attractive approach and has been investigated in animal models and humans. Small molecules which have been identified to block Th17 differentiation are also potential therapeutics in T1D. Monoclonal antibody based therapy targeting IL-17 has been well studied in other autoimmune diseases in humans. Two monoclonal antibodies targeting IL-17 or Th17 approved to treat psoriasis are potentially potent to protect prediabetic individuals from progression into diabetes.

## 2. Th17 Cells and Th17 Cytokines

In 2003, Cua and colleagues [5, 6] demonstrated that IL-23 was crucial for the induction of experimental autoimmune encephalomyelitis (EAE) and collagen-induced arthritis (CIA). IL-23 stimulated IL-17-producing T cells could induce EAE in an adoptive transfer model. Furthermore, mice with a deleted p19 subunit of IL-23 showed significantly reduced number of IL-17-producing T cells and were protected from EAE. In contrast, p35 subunit of IL-12 deficient mice produced an increased number of IL-17-producing T cells and developed severe EAE and CIA. These findings indicated that IL-17-producing T cells driven by IL-23 rather than IFN-*γ*-producing Th1 cells driven by IL-12 are mediating these models of T cell mediated autoimmune diseases. In 2005, two independent groups [1, 2] formally proposed Th17 as a distinct T helper subset and further demonstrated the critical role of Th17 cells in autoimmune diseases. Cytokines required for Th17 cell differentiation and transcription factors governing Th17 cell development are unique and are different from Th1 and Th2 cells [7]. The central modulator of the Th17 lineage is retinoic acid related orphan nuclear receptor (ROR) *γ*t [8] which interacts with other transcription factors in a network to regulate Th17 cells [9, 10]. Th17 cells mainly produce signature cytokine IL-17A (commonly referred to as IL-17), hence its name. However, they also produce IL-17F, IL-21, IL-22, and granulocyte monocyte-colony stimulating factor (GM-CSF) and potentially produce tumor necrosis factor (TNF) and IL-6 [11]. From animal studies in various disease models, IL-17A and IL-17F have shown overlapping but differential functions [12]. The cytokines produced by Th17 cells have broad effects on many cell types and induce the production of proinflammatory cytokines and chemokines, whereas during the process of pathogen clearance, sometimes IL-17-driven inflammation is no longer protective but carries the risk of severe immunopathology and autoimmunity.

IL-17 family cytokines mediate their biological functions via correspondent receptors on the surface of target cells. The IL-17 receptors (IL-17R) constitute a distinct cytokine receptor family [13], which includes IL-17RA, IL-17RB, IL-17RC, IL-17RD, and IL-17RE. Functional receptors for IL-17 family cytokines with IL-17RA as a common subunit often exist in the form of heterodimers. The downstream pathways of IL-17 signaling involve NF*κ*B, MAPKs, and C/EBPs, Act1 being the common membrane proximal adaptor [13].

## 3. Th17 Cells in T1D

Several lines of evidence from studies of animal models of diabetes indicate involvement of Th17 pathway in the pathogenesis of T1D. In spontaneous autoimmune diabetes model in nonobese diabetic (NOD) mice, IL-17A and IL-17F expression in islet correlates with insulitis. Thus, young mice at prediabetic age do not but older diabetic mice do have increased expression of IL-17A or IL-7F in islet along with the development of insulitis [14]. Inhibition of Th17 cells significantly suppressed development of diabetes [15, 16]. IL-17 deficient NOD mice have delayed onset of diabetes with reduced insulitis [17]. In streptozotocin-induced diabetes, IL-23 promotes development of diabetes in subdiabetogenic doses of streptozotocin treatment by expansion of Th17 cells [18]. Moreover, IL-17A deficiency ameliorates streptozotocin-induced diabetes [19]. Purified islet antigen specific Th17 cells are able to transfer diabetes in immunodeficient mice [14, 20]. All these findings clearly demonstrate the critical role of Th17 cells in the development of diabetes. However, as seen in other models of autoimmune diseases, the role of Th17 cells in the pathogenesis and their relation with Th1 cells in mediating the disease in these diabetes models is not of a clear-cut picture. Th1 cells or IFN-*γ* is often associated with increased expression of Th17 cells. Moreover, islet antigen specific Th17 cells need to convert into Th1-like cells to be able to induce diabetes in an adoptive transfer model [14, 20]. IL-17 and interferon- (IFN-) *γ* receptor double knockout mice show significantly delayed onset of diabetes compared to IL-17 single knockout mice [17]. These data suggest that Th17 cells might cooperate with Th1 or IFN-*γ* in mediating inflammation in diabetes. However, IFN-*γ* induced by innocuous islet antigens shows therapeutic effect of diabetes in NOD mice through inhibition of IL-17 production [15]. A recent study provides a novel mechanism for Th17-mediated diabetes which is independent of IFN-*γ* but dependent on TNF [21]. Nonetheless, data from most studies are in favor of an indispensable role of IL-17/Th17 cells in the development of T1D which is supported by the therapeutic effect of IL-17 blockade by anti-IL-17 antibody or IL-25 [16].

Human studies have also generated evidence to support the notion that Th17 cells are critical in the pathogenesis of T1D. Peripheral blood CD4^+^ T cells from new onset T1D children produce higher levels of IL-17, IL-22 and increased* Rorc2* and* Foxp3* gene expression compared with those from healthy individuals upon polyclonal activation, while no increased IFN-*γ* level or T-bet expression was detected in T1D patients. This observation clearly indicates a Th17 biased response in T1D patients. Interestingly, memory CD4^+^ T cells from half of T1D patients show increased IL-17 and IL-22 secretion and* Rorc2* expression* ex vivo* indicating a Th17 response* in vivo* [22]. Similarly, in another study, increased number of IL-17-producing CD4^+^ T cells was also readily detected in new onset T1D children [23]. More importantly, these circulating CD4^+^ T cells in T1D patients produce IL-17 when they are activated by *β*-cell autoantigens including proinsulin, insulinoma-associated protein, and GAD65 peptides [24]. The increased levels of IL-17 in T1D may be attributed to the presence of proinflammatory cytokine milieu that drives toward Th17 differentiation. Indeed, monocytes from T1D patients spontaneously secrete substantially higher levels of IL-6 and IL-1*β* which promote IL-17 production by memory CD4^+^ T cells [25]. More compelling evidence for Th17 biased response in human T1D is provided by Ferraro and colleagues [26]. In response to polyclonal activation, CD4^+^ T cells with memory phenotype from pancreatic-draining lymph nodes (PLN) of T1D patients produce higher levels of IL-17 but not IFN-*γ* or IL-4. Moreover, these PLN memory CD4^+^ T cells release increased levels of IL-17 in response to diabetes-related antigens, proinsulin, and GAD65 [26]. As seen in animal models and in rheumatoid arthritis, IL-17 and IFN-*γ* dual producing cells have also been observed and are emerging preceding clinical diabetes suggesting their involvement in progression to full blown diabetes [27]. In a single case of patient who died 5 days after T1D diagnosis, increased mRNA expression of IL-17A, RORC, and IL-22 along with IFN-*γ* was detected [24] suggesting the direct involvement of IL-17 in destruction of *β* cells and this notion is supported by* in vitro* data where IL-17 has direct toxicity to human *β* cells. Human islet cells express high levels of IL-17RA and IL-17RC. IL-17 alone and in synergy with IFN-*γ* and IL-1*β* increases expression of SOD2, NOS2A, and COX2 which are involved in the inflammatory response in islet cells. Furthermore, IL-17 inhibits BCL-2 gene expression leading to the enhanced proapoptotic effect of IL-1*β*/IFN-*γ* in primary human islet cells [22, 24].

## 4. Th17 and Treg Cells in T1D

Th17 and Treg cells are two closely related CD4^+^ T helper cells subsets in ontogeny. Both Th17 and Treg cells can be differentiated from the same naïve CD4^+^ T cells depending on the presence of different amount of TGF-*β* and absence or presence of proinflammatory cytokines. The homeostasis between Th17 and Treg is important in keeping autoimmunity in check. It is clear that in murine models dysfunction of Treg cells can lead to autoimmune diabetes with increased Th17 cells. Imbalance between Th17 and Treg cells has been noted in several autoimmune inflammatory conditions including T1D [26, 28, 29]. The imbalance is manifested by expansion of Th17 cells which is concomitant with decreased number or function of Treg cells. For example, Ferraro et al. found that expansion of Th17 cells and functional defects in Tregs are key features of the PLN in T1D patients [26]. Earlier studies in human T1D have reported conflicting results with those that T1D patients have decreased [30], increased [31], or equivalent [32–34] numbers of Treg cells compared with healthy individuals. This conflict may stem from poor understanding of the complexity of Foxp3 expressed Treg cells. It has been realized that some IL-17 secreting CD4^+^ T cells also express Foxp3 but do not exert suppressive function. For example, Marwaha et al. demonstrate that new onset T1D children have an increased proportion of CD45RA^−^CD25^int^Foxp3^low^ CD4^+^ T cells secreting high levels of IL-17, which should be identified as effector Th17 cells [23].

The potential mechanisms of how Treg cells regulate Th17 cells response have been explored by several studies. Chaudhry et al. report that CD4^+^ Treg cells control Th17 immune response in mice via Foxp3 binding to STAT3, a key factor in the initiation of Th17 differentiation [35]. Overexpression of Foxp3 results in a strong reduction of IL17A gene expression by inhibiting ROR*γ*t-mediated IL-17A mRNA transcription. This has been shown to be through direct interaction of Foxp3 with ROR*γ*t [36]. These observations support that impaired expression of Foxp3 may lead to defective control of Th17 cells. On the other hand, Th17 cells counteract the Treg cells to expand and allow the development of T1D. Emamaullee et al. found that NOD mice treated with anti-IL-17 could significantly increase the proportion of Foxp3^+^Treg cells [16]. IL-17A and IL-21 induce Th17 and inhibit Tregs redifferentiation via Th17-associated signaling pathway in immune thrombocytopenia patients* in vitro* [37].

## 5. The Interplay between the Gut Microbiota and Th17/Treg Cells in T1D

Both genetic susceptibility and environmental factors are critical in T1D development. Gut microbiota is one of the important environmental factors in development of T1D. Microorganisms inhabiting humans have coevolved in a reciprocal manner with the host to form a status called symbiosis in health state. In particular, microorganisms residing in the mucosal surfaces such as the gut have a profound impact on the human immunity. The human immune system in turn has a great influence on the composition of gut microorganisms [38, 39]. The human gut is colonized with as many as 100 trillion bacteria [40] that are crucial for the development of the immune system, as well as lymphocyte development and their functions. It has been shown that alterations of the gut bacterial composition resulted in changes in T1D onset and progression in several animal models [41–48]. It was thought that studies with germ-free mice will generate definitive data about the role of gut microbiota in development of T1D. However, in contrast to observations in animal models of arthritis and multiple sclerosis, earlier studies reported that germ-free environment exacerbated autoimmune diabetes in NOD mice, but this was not reproduced in recent studies [42, 49]. Since differentiation of Th17 cells and generation of Treg cells are profoundly influenced by gut microbiota, several studies have investigated the effect on Th17 and Treg cells in diabetes models when gut microbiota is modulated. Ivanov et al. first demonstrated the critical role of gut commensal flora in Th17 cell differentiation in antibiotics treated mice [50]. In C57BL/6 mice, broad spectrum antibiotics or vancomycin treatment results in diminished differentiation of Th17 cells in small intestine lamina propria and germ-free environment essentially devoid of Th17 cells with an increased number of Treg cells [50]. These data suggest that composition of gut commensal microbiota regulate the balance of Th17 and Treg cells and may influence intestinal immunity. Later studies identified segmented filamentous bacterium (SFB) being the most important strain of bacteria to induce Th17 differentiation [37, 51], while other commensal bacteria and their metabolites such as short chain fatty acids promote Treg cells [52–54]. Surprisingly, germ-free NOD mice show a slight but significantly increased number of Th17 and Th1 cells in the colon, mesenteric and pancreatic-draining lymph nodes while the Treg cell number is decreased [42]. Consequently, these germ-free NOD mice develop accelerated insulitis although the incidence of diabetes is not changed compared with mice kept in specific pathogen-free (SPF) environment [42]. Whereas data presented above are in favor of a pathogenic role of IL-17/Th17 cells in T1D, recent studies in NOD mice suggested a protective effect of Th17 cells in T1D when gut microbiota is manipulated [55–57]. Biobreeding diabetes prone (BBDP) and biobreeding diabetes resistant (BBDR) rats which are classic models of T1D can provide novel evidence for gut microflora in the context of T1D [46, 58]. It was shown that oral transfer of* Lactobacillus johnsonii* strain N6.2 (LjN6.2) from BBDR to BBDP rats conferred T1D resistance in BBDP rats. The diabetes resistance in LjN6.2-fed BBDP rats was correlated to a Th17 cell bias within the mesenteric lymph nodes and it was concluded that this Th17 cell bias is most likely to contribute to the diabetes protection [56]. In an attempt to resolve the controversy in regard to pathogenic versus protective effects of SFB and Th17 cells on T1D, Kriegel et al. [57] conducted a detailed survey about incidence of diabetes in NOD mice and correlated the incidence with levels of SFB colonization and number of Th17 cells among NOD mice housed in different animal facilities including different facilities in authors' institution and commercial vendors, namely, Jackson and Taconic Farms. In female mice colonized with SFB, the incidence of diabetes is as low as 20%, while 80% of those without SFB colonized develop diabetes at 30 weeks of age. Interestingly, the incidence of diabetes in males remains 10–15% regardless of their SFB colonization status. The number of Th17 cells in the SFB positive mice is correlated with SFB level in the feces. These studies clearly demonstrated correlation of Th17 cells with low incidence of diabetes but do not prove Th17 or IL-17 actually has the protective effect in T1D. The function of these “Th17” was not investigated. It is possible that these “Th17” cells are not the same effector Th17 cells. Indeed, Foxp3^+^/ROR*γ*t^+^IL-17-producing T regulatory cells in T1D in NOD mice have been reported. These regulatory cells migrate to the site of inflammation and protect NOD mice from diabetes [59]. In addition, how host cell signaling pathways are initiated by SFB is not clear. It is possible that SFB influence antimicrobial proteins and molecules expression in epithelium which participate in Th17 cell polarization. And SFB may also act directly on cells of the immune system (reviewed in [38]). Most recently, it is reported that* in vitro* generated IL-17-producing T regulatory cells for BDC2.5 TCR transgenic CD4^+^ T regulatory cells (cultured with TGF-*β* and IL-6) were able to inhibit transfer of diabetes [60]. However, it is not known whether these IL-17-producing CD4^+^ T regulatory cells are induced by SFB. Obviously more studies are required to verify the role of Th17 cells in T1D and their relation to gut microbiome in NOD mice. For example, detailed analysis of cytokine profile of Th17 cells in those NOD mice with SFB is needed; recolonization of germ-free NOD mice with SFB and neutralization of IL-17 activity or deletion of Th17 cells will be required to further clarify this issue.

It must be noted that SFB is not present in humans. Many studies on gut microbiome of T1D children have revealed various results with several species of bacteria in abundance, but these have not been related to Th17 or Treg cell number or function (reviewed in [61]).

## 6. Therapeutic Implications 

At present, the treatment of T1D mainly depends on insulin supplement. However the insulin treatment does not halt disease progression. So the treatment strategies directed on pathogenesis are needed imperiously. Emerging evidence has shown that therapeutic agents targeting the IL-17 or directly inhibiting Th17 cells regulate autoimmune diabetes in NOD mice. For instance, blocking IL-17 activity with anti-IL-17 antibody reduced peri-islet T cell infiltrates and prevented disease in NOD mice. Interestingly, inhibition of Th17 cell differentiation using recombinant IL-25 (IL-17E, a member of IL-17 family cytokine with an anti-inflammatory property and promoting Th2 cell differentiation) had more profound effect than that blocking a single Th17 cytokine, IL-17 using anti-IL-17 antibody in prevention of diabetes. The therapeutic superiority of Th17 cell blockade points out the importance of Th17 cells in the pathogenesis of diabetes and future therapeutic strategy should aim to target Th17 cells rather than targeting individual Th17 cytokines. In light of this view, it is of great interest to evaluate the therapeutic efficacy of recently discovered small molecules which act on ROR*γ*t. For instance, Solt et al. [62] administered SR1001, a selective ROR*α*/*γ* inverse agonist in NOD mice showing significantly reduced diabetes incidence and insulitis in the treated mice. Furthermore, SR1001 reduced proinflammatory cytokine expression, particularly Th17-mediated cytokines, reduced autoantibody production, and increased the frequency of Foxp3^+^ CD4^+^ Treg cells. These data suggest that use of ROR-specific synthetic ligands targeting this cell type may prove utility as a novel treatment for type 1 diabetes (Lin et al., this issue).

Moreover, it was reported that T cell vaccination (TCV) treatment inhibits autoimmune diabetes induced by multiple low-dose streptozotocin (MLD-STZ) in mice through the suppression of intrapancreatic Th17 cells through inhibition of STAT3-mediated RORgt activation [63]. Administration of B7-H4-immunoglobulin fusion protein (B7-H4.Ig), a newly identified T cell coinhibitory signaling molecule, blocks the onset of diabetes in NOD mice [64]. The reduction of diabetes is due to a transient increase of Foxp3^+^ CD4^+^Treg cells at one week posttreatment. Furthermore, the diabetes protection was associated with inhibiting the generation of Th17 cells which subsequently decreases IL-17 production and effectively inhibits the development of T1D in NOD mice [64]. Besides, Bertin-Maghit et al. reported that administration of tolerizing RelB(lo) dendritic cells in 4-week-old NOD mice showed significantly inhibited diabetes progression, which may depend on exacerbating the IL-1-dependent decline in Treg function and promoted Th17 conversion [65]. In addition, ONX 0914 is a selective inhibitor of the immunoproteasome subunit low molecular mass polypeptide (LMP) 7 (*β*5i) that attenuates disease progression in mouse models of diabetes. Immunoproteasome subunit LMP7 inhibition by ONX 0914 suppresses Th1 and Th17 but enhances Treg differentiation [66].

It has been reported that injection of adjuvants that contain mycobacterium such as bacillus Calmette-Guérin (BCG) or complete Freund's adjuvant (CFA) is effective in preventing from the onset of autoimmune diabetes in NOD mice [67]. Recently, Nikoopour et al. demonstrated in an adoptive model that CD4^+^ T cells from CFA-immunized NOD mice which are stimulated with anti-CD3 in the presence of TGF-*β* plus IL-6 or IL-23 can delay diabetes development in recipient mice, suggesting that CFA induces a regulatory Th17 subset [68]. These regulatory Th17 cells produce IL-17, IL-10, and IFN-*γ*. It is reasonable to speculate that the diabetic suppressive effect of these regulatory Th17 cells is mediated by IL-10 and IFN-*γ*. In addition, the above adjuvant treatment may be partially due to suppressing Th17 commitment [69].

Given the importance of gut microbiota in shaping Th17 and Treg cell balance, manipulation of composition of gut microbiota has been investigated for therapy of T1D. Markle et al. [47] report that transfer of adult male gut microbiota to immature female NOD mice shows a robust T1D protection. The diabetes transfer capacity of T cells from female recipients is tested in NOD-SCID mice. Onset of T1D in NOD-SCID mice that received T cells from female mice was significantly delayed. These results suggest that the capacity of T cells in female mice was downregulated by the manipulation of gut microbiota. Studies aiming to investigate whether composition alteration of gut microbiota will influence the balance between Th17 and Treg cells are required. Recently, Shi et al. [70] found that oral administration of* Cordyceps sinensis*, a parasitic fungus used widely in traditional Chinese medicines, resulted in reduction in the overall incidence of diabetes in NOD mice, and this was due to an increase in the ratio of Treg to Th17 cells in the spleen and PLN. It is yet to be determined whether this effect was through alteration of gut microbiota. Diet can alter the composition of gut microbiota and potentially influence Th17 and Treg cells. Indeed, young NOD mice were fed with “antidiabetic” diet (ProSobee infant formula) that abolished inflammatory Th17 cells and IL-23 in the colon and significantly prevented diabetes [70]. This effect is presumably the result of changes in gut microbiota although they were not assessed. Future studies should assess whether strategies to modify gut microbiota will be able to halt or delay the onset of diabetes in high risk populations of healthy individuals.

Targeting IL-17 and Th17 pathway has been approved by FDA in treating psoriasis and psoriatic arthritis. For example, ustekinumab blocking the common subunit p40 of IL-12 and IL-23 has been shown to be highly effective in treating psoriasis. The therapeutic effect of this monoclonal antibody is considered by predominantly blocking IL-23 and subsequently Th17 cells [71, 72]. Secukinumab targeting IL-17A is also recently approved to treat psoriasis [73, 74]. Both antibodies appear to be safe and well tolerated. It would be worthwhile to investigate whether targeting IL-17 or Th17 will be islet protective in those children with islet autoimmunity but not yet diabetic by using these monoclonal antibodies.

## 7. Conclusion

T1D is a T cell mediated autoimmune disorder which targets and destroys insulin producing pancreatic *β* cells. Accumulating evidence gained from animal models and humans closely connects IL-17/Th17 cells in the context of impaired Treg cells/function to the pathology of T1D. Dysbiosis of gut microbiota as one of the important environment factors in T1D has been considered. Several studies have highlighted the importance of gut flora in modulating the mucosal and systemic immune response involved in T1D, specifically in the disease onset and progression. The composition of intestinal microbiota regulates the Th17:Treg balance and may thus influence intestinal and systemic immunity involved in T1D. Studies on manipulation of gut microbiota with diet or medicine are required to assess the effect on Th17 and Treg cells and development and progression of diabetes in populations at high risk. Immunotherapy targeting IL-17/Th17 has achieved high efficacy in other Th17-mediated conditions. Clinical trials with these biologic drugs should be considered to prevent progression in prediabetic populations.
